# Feasibility and accuracy of dual-layer spectral detector computed tomography for quantification of gadolinium: a phantom study

**DOI:** 10.1007/s00330-017-4737-8

**Published:** 2017-01-25

**Authors:** Robbert W. van Hamersvelt, Martin J. Willemink, Pim A. de Jong, Julien Milles, Alain Vlassenbroek, Arnold M. R. Schilham, Tim Leiner

**Affiliations:** 1 Department of Radiology, University Medical Center Utrecht, P.O. Box 85500, 3508 GA Utrecht, The Netherlands; 2 CT Clinical Science, Philips HealthCare, Best, The Netherlands; 3 CT Clinical Science, Philips HealthCare, Brussels, Belgium

**Keywords:** Dual-energy CT, Dual-layer spectral detector CT, Contrast media, Gadolinium, Material decomposition

## Abstract

**Objectives:**

The aim of this study was to evaluate the feasibility and accuracy of dual-layer spectral detector CT (SDCT) for the quantification of clinically encountered gadolinium concentrations.

**Methods:**

The cardiac chamber of an anthropomorphic thoracic phantom was equipped with 14 tubular inserts containing different gadolinium concentrations, ranging from 0 to 26.3 mg/mL (0.0, 0.1, 0.2, 0.4, 0.5, 1.0, 2.0, 3.0, 4.0, 5.1, 10.6, 15.7, 20.7 and 26.3 mg/mL). Images were acquired using a novel 64-detector row SDCT system at 120 and 140 kVp. Acquisitions were repeated five times to assess reproducibility. Regions of interest (ROIs) were drawn on three slices per insert. A spectral plot was extracted for every ROI and mean attenuation profiles were fitted to known attenuation profiles of water and pure gadolinium using in-house-developed software to calculate gadolinium concentrations.

**Results:**

At both 120 and 140 kVp, excellent correlations between scan repetitions and true and measured gadolinium concentrations were found (*R* > 0.99, *P* < 0.001; ICCs > 0.99, CI 0.99–1.00). Relative mean measurement errors stayed below 10% down to 2.0 mg/mL true gadolinium concentration at 120 kVp and below 5% down to 1.0 mg/mL true gadolinium concentration at 140 kVp.

**Conclusion:**

SDCT allows for accurate quantification of gadolinium at both 120 and 140 kVp. Lowest measurement errors were found for 140 kVp acquisitions.

***Key Points*:**

*• Gadolinium quantification may be useful in patients with contraindication to iodine.*

*• Dual-layer spectral detector CT allows for overall accurate quantification of gadolinium.*

*• Interscan variability of gadolinium quantification using SDCT material decomposition is excellent.*

## Introduction

Material decomposition imaging (MDI) using dual-energy computed tomography (DECT) was first described by Hounsfield in 1973 [1]. Different materials, which cannot be distinguished on the basis of attenuation number, can be distinguished with the use of material decomposition algorithms using DECT acquisitions [2–4]. Materials with high atomic numbers, such as iodine (*Z* = 53) and gadolinium (*Z* = 64), show characteristic high attenuation profiles at low energies owing to a substantial contribution of the photoelectric effect to the attenuation [5]. MDI uses these characteristic attenuation profiles to differentiate these contrast agents from other materials. MDI has not been widely applied in clinical practice until recently. Over the past few years several CT vendors have made DECT commercially available for daily clinical practice. Recently a novel DECT technique has become commercially available, which uses a single tube with a dual-layer detector capable of differentiating between low and high energy X-ray photons, and is further investigated in this study.

One of the most widely researched MDI applications is quantitative mapping of iodine distribution in tissues. The resulting maps can be used as a surrogate for tissue perfusion. Early evidence has shown the clinical capability of iodine quantification with DECT at a specified time point for the detection of myocardial [6–12] and pulmonary perfusion defects [13–16]. In addition, DECT iodine mapping is capable of tumour mass characterization and therapy response assessment [17–19]. However, iodine contrast administration, while safe in most patients, is associated with contrast-induced allergic reactions and nephropathy which can cause acute renal dysfunction [20, 21] and significant morbidity and mortality, especially in high-risk patients [22, 23]. In patients with contraindications to iodinated contrast media, gadolinium-enhanced magnetic resonance (MR) angiography can be used as an alternative. However, depending on the indication, MR angiography may have poor diagnostic value compared to (DE)CT angiography. Gadolinium-based CT has been used off-label in higher doses as an alternative for conventional CT angiography with diagnostic image quality [24, 25]. With the use of DECT, higher attenuation can be achieved at low (monochromatic) energies, which could enable the use of much lower gadolinium concentrations [26, 27]. In addition, accurate gadolinium quantification using DECT could allow for a quantitative evaluation of contrast agent distribution in tissue as a surrogate for tissue perfusion using MDI. Therefore, accurate gadolinium quantification combined with increased attenuation could potentially open up the possibility for gadolinium as an alternative contrast agent for DECT imaging in patients with contraindications to iodinated contrast media.

In several studies the feasibility of gadolinium-enhanced DECT has been reported in phantom and animal models [28–31]. These studies described the capability of spectral differentiation and visualisation [28–30] and accuracy of quantification [31] of gadolinium using DECT. However, the accuracy of gadolinium quantification using the novel dual-layer spectral detector CT system (SDCT) is unknown. Therefore, the aim of the current study was to evaluate the feasibility and accuracy of gadolinium quantification using a SDCT system.

## Materials and methods

### Phantom design

**Fig. 1:**
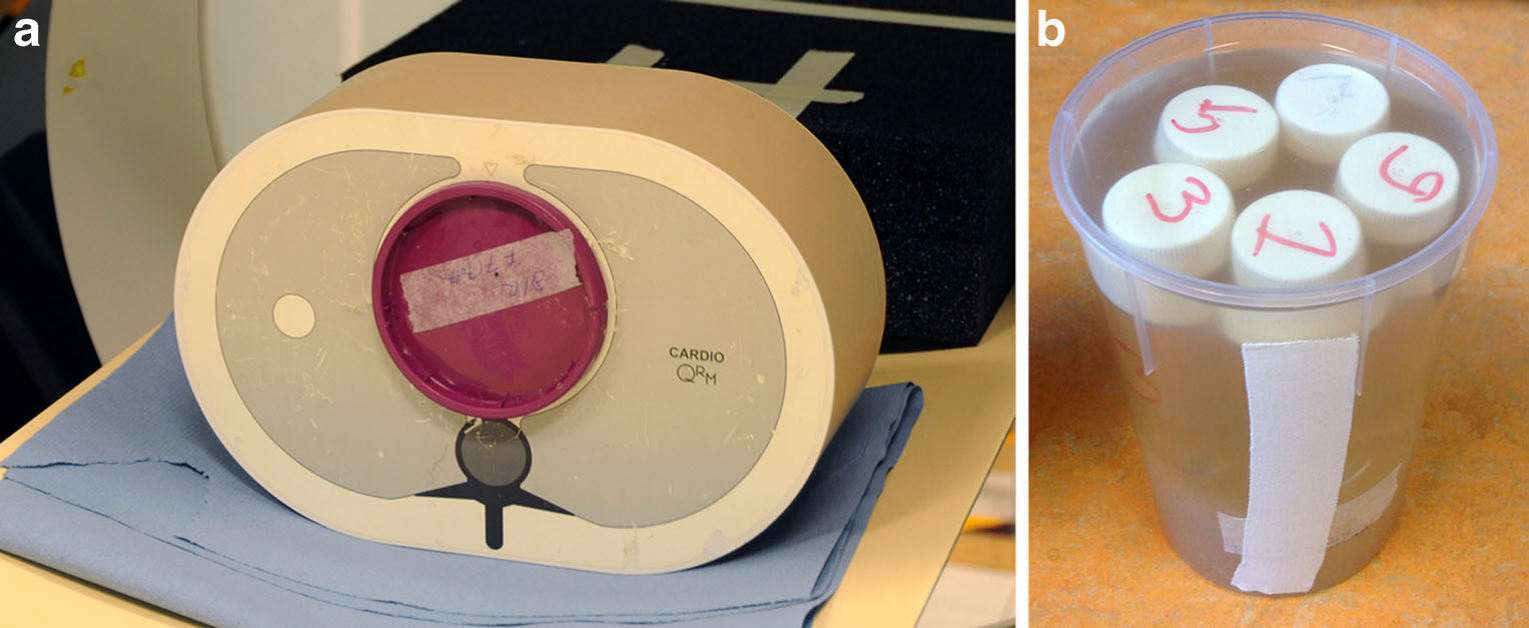
Phantom setup. **a** Anthropomorphic thoracic phantom with a plastic holder placed in the cardiac chamber. **b** Representative plastic holder filled with 5 tubular inserts, with surrounding 2% agar gel solution

An anthropomorphic chest phantom (QRM GmbH, Moehrendorf, Germany) was used. The phantom resembles a chest with corresponding X-ray attenuation behaviour. The phantom has a cylindrical cardiac chamber in which a plastic holder was placed (Fig. 1). Three plastic holders were made, two consisting of five tubular inserts, and one consisting of three tubular inserts with surrounding 2% agar gel solution. In addition, a plastic holder with one tubular insert containing water with surrounding 2% agar gel solution served as control. The fourteen 32-mL tubular inserts contained different concentrations of the gadolinium-based contrast agent gadobutrol (Gadovist 1.0, Bayer Healthcare, Berlin, Germany). One millilitre of this contrast agent contains 157.25 mg gadolinium. Different amounts of gadobutrol were diluted in water, resulting in the following concentrations of gadolinium: 0.0, 0.1, 0.2, 0.4, 0.5, 1.0, 2.0, 3.0, 4.0, 5.1, 10.6, 15.7, 20.7 and 26.3 mg/mL, which is equivalent to 0.000, 0.001, 0.002, 0.002, 0.003, 0.006, 0.013, 0.019, 0.026, 0.032, 0.068, 0.100, 0.132 and 0.167 mmol/mL, respectively.

Concentrations were chosen to mimic an estimated clinical range of gadolinium concentrations encountered after injection of 0.1–0.2 mmol of gadolinium per kilogram. Strich et al. [32] measured percentage dose of gadolinium-based contrast agent per gram of tissue in healthy rabbit organs 5 min after admission, resulting in the following percentages: 0.052%/g heart, 0.073%/g lungs, 0.037%/g liver, 0.037%/g spleen and 0.250%/g kidney. On the basis of these percentages, an estimation of gadolinium concentrations encountered at each organ can be calculated. At 31.5 mg/kg bodyweight (equal to 0.2 mmol/kg) gadolinium administration, a human subject of 70 kg would receive a total of 2201.5 mg gadolinium. On the basis of the percentages determined by Strich and colleagues, gadolinium distribution in the heart 5 min after injection would be 0.00052 × 2201.5 mg, or 1.15 mg per gram myocardium. Myocardial muscle has a specific gravity of 1.05 g/mL [33], implicating an estimated gadolinium concentration encountered in the myocardium of 1.15 mg/g × 1.05 g/mL, or 1.21 mg/mL. Using the aforementioned distribution percentages these calculations can also be applied to the lungs, liver, spleen and kidney, with a calculated estimated specific gravity (weight/volume) of 1.34, 1.01, 0.71 and 0.85 g/mL, respectively [34–36]. Thus, it is to be expected that gadolinium concentrations of 2.15, 0.82, 0.58 and 4.67 mg/mL are encountered in healthy lung, liver, spleen and kidney tissue, respectively. These concentrations are in the range of concentrations evaluated in this study. As it is expected that in tissue with a perfusion defect lower concentrations of gadolinium will be encountered, we also evaluated ultra-low concentrations of gadolinium down to 0.1 mg/mL.

### Image acquisition

Images were acquired using the newest generation 64-detector row SDCT system (iQon Spectral CT, Philips Healthcare, Best, the Netherlands). This system uses a single X-ray tube and a dual-layer detector. The detector separates the X-ray beam into low (upper layer) and high (lower layer) energy data, which is used to reconstruct spectral-based images (SBI). The SBI contain the raw data of both layers and are used to reconstruct any dual-energy image and/or analysis. In addition, by combining the output of both layers, a conventional image is reconstructed from the data. The phantom was imaged in spiral mode at 120 and 140 kVp. The tube current–time product was set to a fixed value of 200 mAs, resulting in a volumetric CT dose index (CTDI_vol_) of 18.4 and 26.5 mGy for 120 and 140 kVp acquisitions, respectively. The following parameters were used: detector collimation 64 × 0.625 mm, rotation time 0.4 s and pitch 1.046. At both tube voltages, acquisitions were repeated five times with small displacements between each acquisition to take into account interscan variation. Thus, the phantom was translated a few millimetres in the left–right direction, as well as along the z-axis of the CT scanner. After the five repetitions, the phantom was set back to the starting position.

### Image reconstruction

The raw projection data from both detector layers were automatically reconstructed into SBI. Subsequently, MDI was performed in the projection domain, which efficiently eliminates beam hardening artefacts [37]. All images were reconstructed with standard chest reconstruction filter B and spectral level 3. Spectral is a model-based iterative reconstruction developed for the SDCT, it is an equivalent to iterative model-based reconstruction (IMR). Spectral consists of six levels, whereby a higher spectral level implies more noise reduction. Slice thickness and increment were both 1 mm. The reconstructed images were evaluated on a dedicated workstation using the Spectral CT Viewer (IntelliSpace Portal v6.5.0.02080, Philips Healthcare, Best, the Netherlands).

### Image analysis and gadolinium quantification

**Fig. 2:**
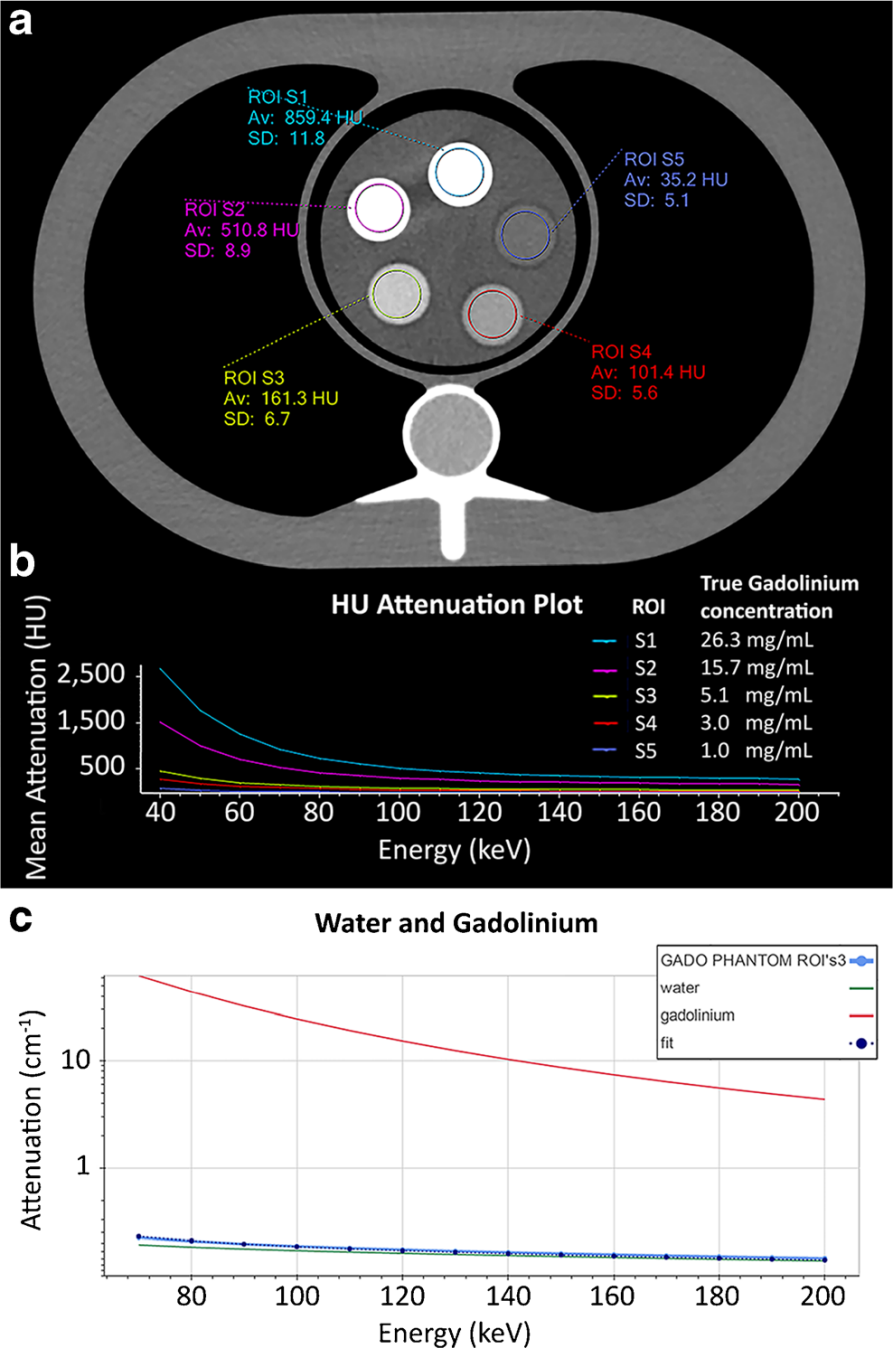
Axial CT image and measurements. **a** Axial conventional SDCT image of the phantom with 5 tubular inserts, surrounded by 2% agar gel. ROIs with a fixed area of 225 mm^2^ drawn in the centre of each insert. **b** A spectral plot of each ROI was conducted, showing mean Hounsfield units plotted against energy in keV. Hounsfield unit values of the spectral plots were extracted in increments of 10 keV. **c** Using in-house-developed software, we reconstructed attenuation profiles between 70 to 200 keV from the extracted Hounsfield units, and a combination of known attenuation profiles of pure gadolinium and water was fitted to the reconstructed attenuation profile. This case concerns ROI S3, containing 5.1 mg of gadolinium per millilitre

On three different slices of each data set a region of interest (ROI) with a fixed size of 225 mm^2^ was drawn in the centre of each insert (Fig. 2a). Subsequently spectral plots of every ROI were obtained, in which mean Hounsfield units (HU) were plotted as a function of different energy levels expressed in kilo electron volt (keV) (Fig. 2b). These mean HU values of the spectral plots were extracted in steps of 10 keV and used as an input for the analysis. The currently used SDCT system uses traditional integrated detectors at two energy spectra and is therefore not able to image and/or quantify a material-specific K-edge [37]. Materials with a K-edge within the SDCT range (40–200 keV) will not show a discontinuity in their attenuation function on the SDCT spectral plot. When evaluating the mean attenuation across monochromatic energies, this does not pose a problem and therefore the whole energy spectrum can be used (40–200 keV). However, for the quantitative analyses of gadolinium concentrations a comparison is made with the attenuation profile of pure gadolinium which does contain the discontinuity in their attenuation function at the K-edge. Therefore, to take into account the non-linear energy dependency close to the K-edge of gadolinium (50.2 keV), only the energy range from 70 to 200 keV was used for the quantitative analysis. With in-house-developed software, attenuation profiles were reconstructed from the provided mean HU, and gadolinium concentrations were calculated by fitting combinations of known attenuation profiles of pure gadolinium and water to the reconstructed attenuation profiles (Fig. 2c). For each ROI drawn in the phantom, the in-house-developed software assumed that all voxels within this ROI were composed of only gadolinium and water and that the sum of these fractions added up to 100%. Known attenuation profiles of pure gadolinium and water were obtained from the National Institute of Standards and Technology (NIST) database [38]. Therefore, no calibration scans with water and/or gadolinium concentrations were needed. For all thirteen different gadolinium concentrations, 15 measurements were performed at both 120 and 140 kVp (three slices, five repetitions). In addition, 30 measurements (15 at both 120 and 140 kVp) were performed on the control phantom. Gadolinium concentrations were calculated for each measurement.

### Attenuation coefficient

**Fig. 3:**
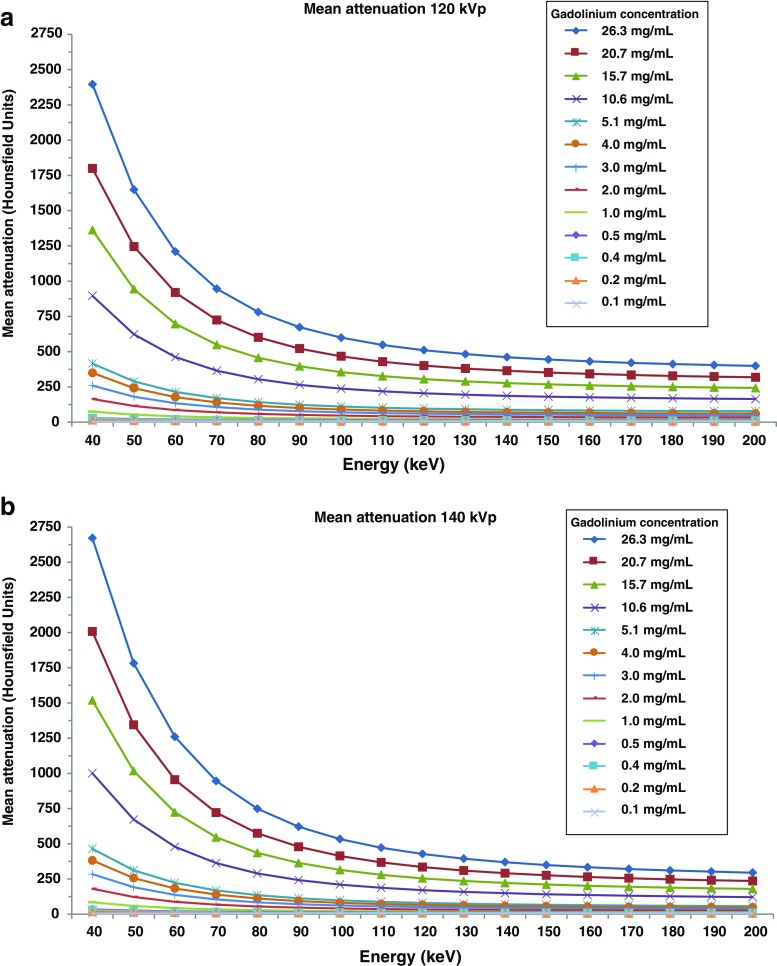
Mean CT attenuation coefficients across all monochromatic energies. Mean CT attenuation of all measurements for each gadolinium concentration, constructed in steps of 10 keV. Graphs were used to investigate the ability of SDCT low monochromatic energies to visually identify an increase in HU values due to the presence of gadolinium-containing contrast media. Scans were performed at 120 kVp (**a**) and 140 kVp (**b**). For subsequent gadolinium quantification, only attenuation profiles between 70 to 200 keV were used for the in-house-developed software analyses (Fig. 2c)

CT attenuation during injection of low gadolinium concentrations (i.e. 0.1–0.2 mmol/kg bodyweight) will generally lead to lower HU values compared to the use of iodinated contrast agents [24, 25] . To investigate the ability of SDCT to visually identify an increase in HU values due to the presence of a gadolinium-containing contrast agent we extracted mean attenuation coefficients across monochromatic energies (40–200 keV) for the different gadolinium concentrations used in this study (Fig. 3).

### Statistical analysis

To evaluate the quantification accuracy of gadolinium concentrations, we defined measurement errors in milligrams per millilitre and relative measurement errors in percentages. Measurement errors were calculated by subtracting true gadolinium concentrations from the measured gadolinium concentrations. Subsequently, relative measurement errors (%) were calculated as follows:

$$ \mathrm{Relative}\kern0.75em \mathrm{measurement}\kern0.5em \mathrm{error}\left(\%\right)=\frac{\mathrm{measurement}\kern0.5em \mathrm{error}\left(\frac{\mathrm{mg}}{\mathrm{mL}}\right)}{\mathrm{true}\kern0.5em \mathrm{gadolinium}\kern0.5em \mathrm{concentration}\left(\frac{\mathrm{mg}}{\mathrm{mL}}\right)}\times 100\left(\%\right) $$

All measurement error analyses were performed separately for 120 and 140 kVp. In addition, sub-analyses were done for each concentration. The Shapiro–Wilk test was used to identify normally distributed data. For each concentration, statistical differences of measurement errors between 120 and 140 kVp were analysed using paired *t* test for normally distributed data. A Bonferroni corrected *P* < 0.004 (0.05/number of comparisons) was considered significant. Pearson’s correlation coefficient was used to evaluate correlations between measured and true gadolinium concentrations at different tube voltages and for each scan repetition. In addition, reproducibility was evaluated. To define agreement of results, the two-way random single measure intraclass correlation coefficient (ICC) with corresponding confidence interval (CI) was used for all possible two-way interactions. ICCs between 0.61 and 0.80 were considered good and ICCs greater than 0.80 excellent [39]. Measurement interscan variabilities of all scan repetitions were plotted in one single plot by using a modified Bland–Altman plot described by Jones et al. [40]. In this figure the measurement differences of every measurement compared to the mean measurement of all scans are plotted against the mean measurement of all scans. As described by Jones et al., the limits of agreement were calculated as mean ± 1.96 × SD, where the SD is an estimate of the standard deviation for all observers [40]. Values are listed as mean with standard deviation (SD), unless stated otherwise. A *P* value less than 0.05 was used to indicate statistical significance. IBM SPSS version 21.0 (IBM corp., Armonk, New York, USA) was used for statistical analyses.

## Results

Measurements of the water-filled insert, which served as control, yielded 0.0 ± 0.0 mg/mL with a measurement error of 0.0 ± 0.0 mg/mL for all measurements. To avoid influence on measurement accuracy, these control measurements were not included in further statistical analyses.

### Accuracy and reproducibility

**Fig. 4:**
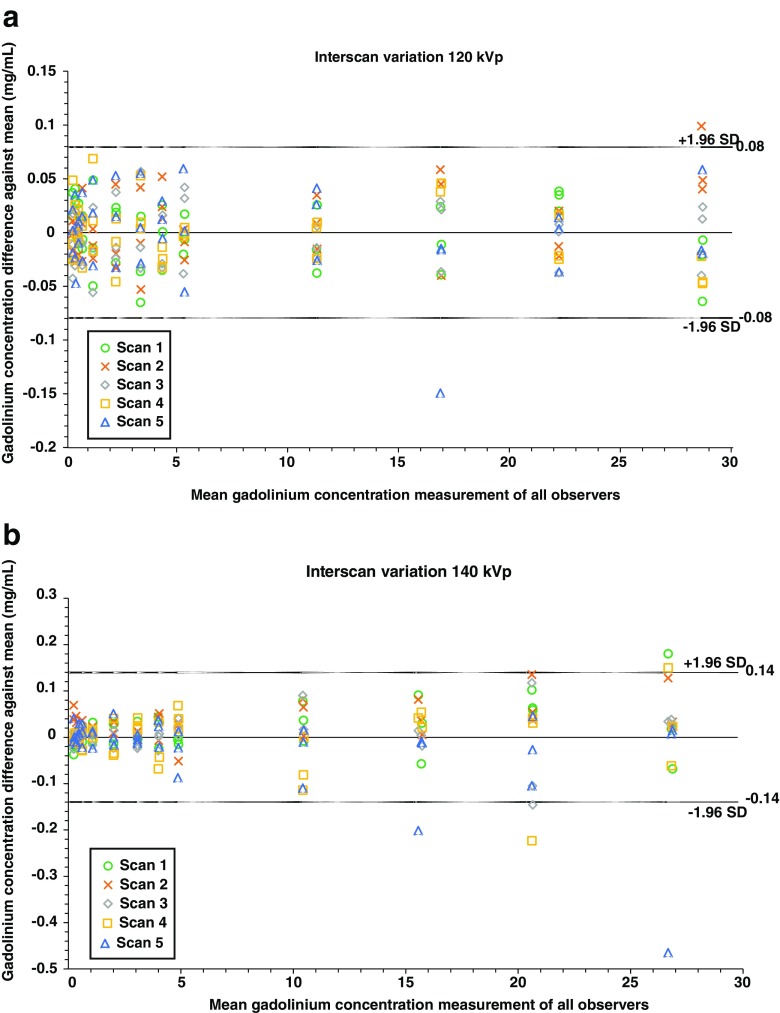
Interscan agreement for all scan repetitions at 120 kVp (**a**) and 140 kVp (**b**). Values are plotted according to Jones et al. [40]. The measurement difference of each scan compared to the mean measurement of all scans is plotted against the mean measurement of all scans

At both 120 and 140 kVp, excellent correlations (*R* > 0.99, *P* < 0.001; ICCs > 0.99, CI 0.99–1.00) were found between true and measured gadolinium concentrations for each scan repetition. In addition, reproducibility between all scan repetitions was excellent (*R* > 0.99, *P* < 0.001; ICCs > 0.99, CI 0.99–1.00). The interscan agreement is displayed in Fig. 4a for 120 kVp and Fig. 4b for 140 kVp. Because excellent correlations were found, all scan repetitions were analysed combined together in subsequent analyses.

### 120 kVp

**Fig. 5:**
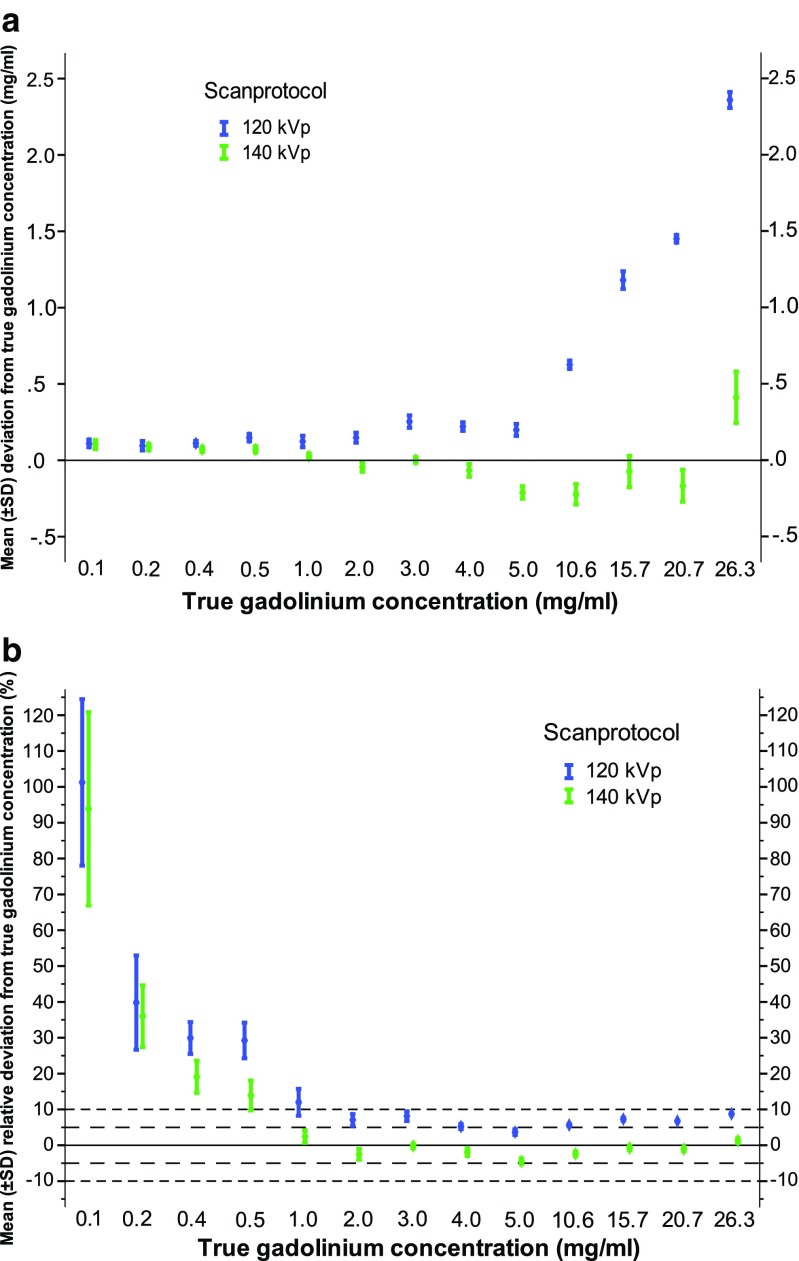
Accuracy of gadolinium quantification. Accuracy expressed as mean measurement error (**a**) and mean relative measurement error (**b**). *Symbol* represents mean and *error bar* the standard deviation

All gadolinium concentrations were overestimated. Mean measurement errors for the 15 ROIs per concentration ranged between 0.1 and 2.4 mg/mL (Table 1, Fig. 5a). For each concentration, measurement errors at 120 kVp were significantly (Bonferroni *P* < 0.004) higher compared to measurement errors at 140 kVp, except for the lowest two concentrations of 0.1 and 0.2 mg/mL. Relative measurement errors (%) were below 10% down to 2.0 mg/mL true gadolinium concentrations and increased up to 29.4% at 0.5 mg/mL and 100.9% at 0.1 mg/mL true gadolinium concentration (Table 1, Fig. 5b).

**Table 1:** Mean errors of gadolinium concentration measurements with a dual-layer spectral detector CT scanner

True concentration (mg/mL)	120 kVp		140 kVp	
	Measurement error		Measurement error	
	mg/mL	%	mg/mL	%
26.3	2.4 ± 0.1*	9.0 ± 0.2	0.4 ± 0.2	1.6 ± 0.6
20.7	1.5 ± 0.0*	7.0 ± 0.1	–0.2 ± 0.1	–0.8 ± 0.5
15.7	1.2 ± 0.1*	7.5 ± 0.4	–0.1 ± 0.1	–0.5 ± 0.7
10.6	0.6 ± 0.0*	5.9 ± 0.3	–0.2 ± 0.1	–2.1 ± 0.6
5.1	0.2 ± 0.0*	3.9 ± 0.8	–0.2 ± 0.0	–4.2 ± 0.8
4.0	0.2 ± 0.0*	5.5 ± 0.7	–0.1 ± 0.0	–1.6 ± 1.0
3.0	0.3 ± 0.0*	8.4 ± 1.3	0.0 ± 0.0	0.1 ± 0.6
2.0	0.1 ± 0.0*	7.3 ± 1.6	–0.0 ± 0.0	–2.2 ± 1.5
1.0	0.1 ± 0.0*	12.1 ± 3.7	0.0 ± 0.0	2.7 ± 1.7
0.5	0.1 ± 0.0*	29.4 ± 5.0	0.1 ± 0.0	14.1 ± 4.1
0.4	0.1 ± 0.0*	30.0 ± 4.4	0.1 ± 0.0	19.3 ± 4.5
0.2	0.1 ± 0.0	39.8 ± 13.1	0.1 ± 0.0	36.1 ± 8.5
0.1	0.1 ± 0.0	100.9 ± 23.1	0.1 ± 0.0	93.5 ± 26.8
0.0	0.0 ± 0.0		0.0 ± 0.0	

Data are given as mean ± standard deviation. For each true concentration 15 measurements were done at both 120 and 140 kVp

*Significantly (Bonferroni *P* < 0.004) higher compared to measurement error at 140 kVp

### 140 kVp

Per concentration (*N* = 15), mean measurement errors varied from −0.2 to 0.4 mg/mL (Table 1, Fig. 5a). Relative measurement errors (%) stayed below 5% down to 1.0 mg/mL true gadolinium concentration. At true gadolinium concentrations between 0.1 and 0.5 mg/mL, mean measurement errors were low with 0.1 ± 0.0 mg/mL deviation; expressed in percentages this varied between 93.5 ± 26.8% and 14.1 ± 4.1% deviation, respectively (Fig. 5b).

### Attenuation coefficient

Overall mean attenuation increased when lowering keV (Fig. 3). At the lowest possible monochromatic energy (40 keV), mean attenuation for the estimated clinical gadolinium range of 0.5, 1.0, 2.0, 3.0, 4.0 and 5.1 mg/mL yielded 28, 74, 164, 260, 349 and 416 HU at 120 kVp and 34, 84, 180, 284, 382 and 464 HU at 140 kVp, respectively.

## Discussion

This study showed that it is feasible to quantify a commonly clinically encountered range of gadolinium concentrations in a phantom model with overall high accuracy and reproducibility using an in-house-developed material decomposition method on a novel clinical dual-layer spectral detector CT system.

Whereas conventional CT displays anatomical structures as a function of tissue density, DECT enables enhanced tissue characterization using MDI. Quantitative assessment of contrast agent uptake and its provided distribution map can be used as a surrogate for tissue perfusion [6–12, 14]. In the current study we showed that clinically encountered low concentrations of gadolinium, down to 0.5 mg/mL, can be accurately quantified with a mean measurement error of 0.1 mg/mL using SDCT at both 120 and 140 kVp. In the ultra-low gadolinium concentration range (0.1–0.4 mg/mL), expected to be encountered in tissues with a perfusion defect, the mean measurement error remained around 0.1 mg/mL at both 120 and 140 kVp. However, at these low concentrations the margin of error increased substantially and approached the gadolinium concentration itself, indicating that the lower limit of reasonably accurate gadolinium quantification using SDCT lies between 0.5 and 1.0 mg/mL. In the range of clinically encountered gadolinium concentrations (0.5–5.1 mg/mL) after administration of 0.1–0.2 mmol/kg bodyweight, mean CT numbers at 40 keV ranged between 28 and 464 HU (Fig. 3). The combination of high(er) attenuation at lower monochromatic energies and accurate quantification of low gadolinium concentrations opens up the possibilities for DECT scanning with the use of gadolinium as a contrast agent. Potential clinical applications include detection of myocardial [6–12] and pulmonary perfusion defects [14–16] and the characterization of tumour masses and therapy response assessment [17–19].

In clinical routine, adequate tissue contrast and contrast agent density maps are important for the diagnosis and evaluation of organ perfusion defects. However, to be able to create a gadolinium density map as a surrogate for tissue perfusion, accurate gadolinium quantification is essential, as the post-processing is based on these measurements. This is the first study to describe the accuracy of gadolinium quantification using MDI on SDCT. Gabbai et al. [28] described the capability of spectral differentiation of gadolinium using SDCT, which is in accordance with our study. However, no quantitative values were described and high concentrations (4.7–187.6 mg/mL) of gadolinium were used, which is at least one to two orders of magnitude above the estimated range encountered in healthy cardiac, lung, liver, spleen and kidney tissue (0.58–4.66 mg/mL). Zhang et al. [30] showed a high sensitivity and specificity for gadolinium-enhanced dual-source DECT pulmonary angiography to detect pulmonary embolism in rabbits. However, as in the study by Gabbai et al. gadolinium concentration was not quantified. In addition, high intravenous doses of gadolinium contrast agent, 1.5 and 2.5 mmol/kg bodyweight, were administrated. Bongers et al. [31] evaluated the potential of gadolinium as a CT contrast agent using dual-source DECT in a phantom setup. In accordance with our study they found that monochromatic images at low energy (e.g. 40 keV) allow for higher attenuation. Additional quantification was performed by using the material-specific dual-energy ratio for gadolinium. For the true gadolinium concentrations 6.3, 3.2, 1.6, 0.8, 0.4 and 0.2 mg/mL relative measurement errors were 11.5, 12.0, 21.6, 21.6, 104.2 and 159.4%, respectively. In our study we found a higher accuracy with relative measurement errors of less than 10% down to 2.0 mg/mL at 120 kVp and 1.0 mg/mL at 140 kVp. A possible explanation for this difference can be found in the algorithm. The post-processing algorithms used by Bongers et al. [31] was originally designed for iodine, whereas our algorithm was specifically designed for gadolinium quantification.

We found a slightly lower measurement error, and thus higher accuracy, for scans acquired at 140 kVp compared to 120 kVp. When scanning with a higher tube voltage, more high energy X-ray photons are produced. This decreases the spectral overlap between high- and low-energy spectra, and thereby improves the accuracy of material decomposition, which is in accordance with the findings of Gabbai and colleagues [28]. Moreover, 140 kVp acquisitions resulted in higher CT numbers of different gadolinium concentrations at monochromatic 40 keV images (34–464 HU) compared to 120 kVp acquisitions (28 to 416 HU), indicating a superior spectral separation at a higher tube voltage.

Even though gadolinium chelates are generally considered to be safe contrast agents, with acute reaction rates of approximately 0.001–0.07% [41], recently concerns have arisen about their long-term safety after the discovery that administration of multiple doses has led to detectable gadolinium levels in the brain [42, 43]. In addition, gadolinium contrast has been linked to an increased risk of nephrogenic systemic fibrosis (NSF) in patients with impaired renal function [44]. In both conditions the linear non-ionic and linear ionic contrast agents have primarily been implicated, whereas macrocyclic gadolinium agents, such as used in the current study, have not been linked conclusively to either of these conditions [45–47]. Although both iodine and gadolinium contrast agents pose a risk for patients with impaired renal function, gadolinium is thought to be preferred in patients with renal failure and a glomerular filtration rate greater than 30 mL/min since the risk of NSF is low in these patients, while the risk of iodine contrast-induced nephropathy clearly exists [41]. Furthermore, using gadolinium could potentially obviate the need for pre- and post-imaging hydration as well as premedication protocols that are commonly used in patients with impaired renal function who undergo contrast-enhanced CT scanning, or patients with known allergies to iodinated contrast agents. In the current study a relatively simple method for material decomposition using in-house-developed software is proposed. Our method is based on the mass attenuation coefficient across monochromatic energies. Monochromatic reconstructions take into account the function of two independent factors: the photoelectric and the Compton effect [2]. The photoelectric effect is strongly related to the atomic number of a material in the CT energy range and is therefore material-specific [37]. Our method takes into account this material-specific effect by evaluating the attenuation across monochromatic energies.

The strength of our study is that we evaluated accuracy of gadolinium quantification in an optimal controlled setting with a wide and clinically relevant range of gadolinium concentrations, which provides the basis for further research and clinical applications. Our study also has some limitations. The most important is that we used a static phantom in which organ motion was not taken into account. In addition, a fixed concentration is not the same as a bolus injection. However, we tried to mimic the clinical situation as best as possible by using low concentrations of gadolinium, which are expected to be typically encountered clinically. A second limitation is that our study only takes into account water and gadolinium when calculating the amount of gadolinium concentration. Since human tissue does not only consist of water and gadolinium, future phantom and patient research will have to address (healthy) tissue attenuation as well using a three- or multi-material decomposition method. A third limitation is the need for relatively high peak tube voltage (120 or 140 kVp) settings to ensure sufficient spectral separation. However, the higher radiation dose due to the use of high kVp acquisitions can be addressed by reducing tube current (mAs). The fourth limitation is that we only evaluated one DECT technique; therefore, our results may be limited to the vendor used in this study.

In conclusion, SDCT allows for accurate quantification of commonly clinically used gadolinium concentrations at both 120 and 140 kVp. Lowest measurement errors were found for 140 kVp acquisitions.

## Abbreviations

CI: Confidence interval
CT: Computed tomography
DECT: Dual-energy computed tomography
HU: Hounsfield units
ICC: Intraclass correlation coefficient
keV: Kilo electron volt
kVp: Kilovoltage peak
mAs: Milliampere second
MDI: Material decomposition imaging
MR: Magnetic resonance
NIST: National Institute of Standards and Technology
ROI: Region of interest
SBI: Spectral-based images
SDCT: Dual-layer spectral detector computed tomography
